# Thermal regime and host clade, rather than geography, drive *Symbiodinium* and bacterial assemblages in the scleractinian coral *Pocillopora damicornis sensu lato*

**DOI:** 10.1186/s40168-018-0423-6

**Published:** 2018-02-20

**Authors:** Kelly Brener-Raffalli, Camille Clerissi, Jeremie Vidal-Dupiol, Mehdi Adjeroud, François Bonhomme, Marine Pratlong, Didier Aurelle, Guillaume Mitta, Eve Toulza

**Affiliations:** 1 IHPE, UMR 5244, University of Perpignan Via Domitia, CNRS, IFREMER, University of Montpellier, Perpignan, France; 2 ENTROPIE, UMR 9220 & Laboratoire d’Excellence CORAIL, IRD, University of Perpignan Via Domitia, Perpignan, France; 3 ISEM, UMR 5554, CNRS, University of Montpellier, IRD, EPHE, Sète, France; 4 IMBE, UMR 7263, Aix Marseille University, CNRS, IRD, Avignon University, Marseille, France

**Keywords:** Coral holobiont, Microbiota, Bacterial communities, *Symbiodinium* assemblages, Thermal adaptation, Scleractinian corals, Coral reefs, *Pocillopora damicornis*

## Abstract

**Background:**

Although the term holobiont has been popularized in corals with the advent of the hologenome theory of evolution, the underlying concepts are still a matter of debate. Indeed, the relative contribution of host and environment and especially thermal regime in shaping the microbial communities should be examined carefully to evaluate the potential role of symbionts for holobiont adaptation in the context of global changes. We used the sessile, long-lived, symbiotic and environmentally sensitive reef-building coral *Pocillopora damicornis* to address these issues.

**Results:**

We sampled *Pocillopora damicornis* colonies corresponding to two different mitochondrial lineages in different geographic areas displaying different thermal regimes: Djibouti, French Polynesia, New Caledonia, and Taiwan. The community composition of bacteria and the algal endosymbiont *Symbiodinium* were characterized using high-throughput sequencing of 16S rRNA gene and internal transcribed spacer, ITS2, respectively. Bacterial microbiota was very diverse with high prevalence of *Endozoicomonas*, *Arcobacter*, and *Acinetobacter* in all samples. While *Symbiodinium* sub-clade C1 was dominant in Taiwan and New Caledonia, D1 was dominant in Djibouti and French Polynesia. Moreover, we also identified a high background diversity (i.e., with proportions < 1%) of A1, C3, C15, and G *Symbiodinum* sub-clades. Using redundancy analyses, we found that the effect of geography was very low for both communities and that host genotypes and temperatures differently influenced *Symbiodinium* and bacterial microbiota. Indeed, while the constraint of host haplotype was higher than temperatures on bacterial composition, we showed for the first time a strong relationship between the composition of *Symbiodinium* communities and minimal sea surface temperatures.

**Conclusion:**

Because *Symbiodinium* assemblages are more constrained by the thermal regime than bacterial communities, we propose that their contribution to adaptive capacities of the holobiont to temperature changes might be higher than the influence of bacterial microbiota. Moreover, the link between *Symbiodinium* community composition and minimal temperatures suggests low relative fitness of clade D at lower temperatures. This observation is particularly relevant in the context of climate change, since corals will face increasing temperatures as well as much frequent abnormal cold episodes in some areas of the world.

**Electronic supplementary material:**

The online version of this article (10.1186/s40168-018-0423-6) contains supplementary material, which is available to authorized users.

## Background

All partners (bionts) involved in a stable symbiosis, and thus being part of the entire organism, constitute the holobiont [1]. A decade after this term has been defined; it has been popularized in corals [2] and subsequently led to the hologenome theory of evolution [3–6]. The hologenome is defıned as the sum of the genetic information of the host and its symbiotic microorganisms. In this context, phenotypes are the product of the collective genomes of the holobiont partners, being the true unit of biological organization and thus the object of natural selection [7–9]. This concept has gained increased attention for many issues on the functioning, homeostasis, or evolution of living organisms, extending our knowledge of microbial community associated to them (see [10] for a review on metaorganisms).

Scleractinian corals (the major reef-building organisms of coral reef ecosystems) are considered as the most diverse symbiotic ecosystem studied to date [11], forming a complex consortium composed by the cnidarian host, as well as microbial eukaryotes (including the dinoflagellate endosymbiont *Symbiodinium*), prokaryotes (bacteria and archae), and viruses. The symbiosis between corals and dinoflagellate algae of the genus *Symbiodinium* provides the foundation for the ecological success of coral reefs over millions of years [12]. In this phototrophic and potentially mutualistic association, the coral host provides inorganic nutrients in exchange of photosynthetically fixed carbon (photosynthates) and amino acids from the algal symbiont [12–14]. Algae from the *Symbiodinium* genus are classified into nine clades (from A to I) [15, 16], and the physiology of the coral holobiont is affected by the clade of the symbiont [17, 18]. Although the establishment of a specific symbiosis occurs during early stages of host larvae colonization [19], some coral species can switch endosymbiotic algae during their lifetime in response to major environmental stressors such as thermally induced bleaching events [20, 21], although the long-term persistence of such changes is matter of debate [22]. Bacterial communities associated to corals have also been extensively studied comparing different species [2, 23], disease states [24–26], or environmental conditions [27, 28] (see also [3] for a review).

Despite this large corpus of studies, none addressed the effect of natural thermal regimes on microbial assemblages in scleractinian corals. However, sea surface temperature increase is the main factor of ongoing climate changes affecting reef-building corals (32.8% of species being considered at risk of extinction) [29] with mass mortalities following severe and recurrent bleaching events [30].

In this study, we investigated the effect of thermal regimes, as well as host clade and geographical distribution, on the bacterial and *Symbiodinium* assemblages in the complex *Pocillopora damicornis sensus lato* (Veron and Pichon 1976), a functional group of environmentally sensitive scleractinian corals [31] that was recently split into five clades [32–34]. High-throughput metabarcoding allowed us to access for the first time the unculturable diversity of both bacterial communities and *Symbiodinium* assemblages in coral populations from different locations with contrasting thermal regimes.

## Methods

### Sampling sites and study design

Colonies of *Pocillopora damicornis sensus lato* growing between 1 and 5 m depth were sampled by snorkeling from four regions (Djibouti, Taiwan, New Caledonia, and French Polynesia [35]) in 15 localities (Table 1). A total of 94 colonies were sampled during this survey. The tip (1–2 cm) from one healthy branch of each colony was cut and disposed individually in a plastic bag held in seawater during the sampling cruise. Samples were subsequently transferred into modified CHAOS buffer (4 M guanidium thiocyanate, 0.5% *N*-lauryl sarcosine sodium 25 mM Tris-HCl pH 8, 0.1 M b-mercaptoethanol) as described [36].

**Table 1:** Details of the sampling of *Pocillopora damicornis sensu lato* conducted in this study

Region	Locality	Code	Latitude	Longitude	Nb
Djibouti	Moucha Island	DJMI	11° 43′ 49.27″ N	43° 13′ 26.69″ E	7
	Ras Korali	DJRK	11° 46′ 46.15″ N	42° 55′ 27.65″ E	8
	Sable Blanc	DJSB	11° 34′ 57.41″ N	42° 47′ 45.86″ E	12
French Polynesia	Moorea island—Papetoai	FPMP	17° 29′ 31.72″ S	149° 52′ 8.34″ O	6
	Moorea island—Tiahura	FPMT	17° 29′ 22.90″ S	149° 53′ 48.65″ O	5
	Raiatea island—Avera	FPRA	16° 47′ 22.00″ S	151° 23′ 30.63″ O	8
	Tahaa island—Tapuamu	FPTT	16° 36′ 51.03″ S	151° 32′ 33.91″ O	5
	Tahaa island—Vaitoare	FPTV	16° 40′ 36.26″ S	151° 27′ 18.92″ O	9
	Tahiti island—Papeete	FPTP	17° 34′ 27.93″ S	149° 37′ 11.07″ O	7
New Caledonia	Baie de Ste. Marie	NCBS	22° 17′ 35.86″ S	166° 28′ 14.44″ E	4
	Baie de Ma’a	NCBM	22° 11′ 45.17″ S	166° 20′ 23.67″ E	4
	Grande Rade	NCGR	22° 14′ 38.63″ S	166° 25′ 7.58″ E	6
Taiwan	Outlet	TWOU	21° 55′ 48.4″ N	120° 44′ 41.9″ E	6
	Hobihu	TWHO	21° 56′ 18.6″ N	120° 44′ 45.7″ E	4
	Wanliton	TWWA	21° 59′ 37.7″ N	120° 42′ 22.7″ E	3

*Nb* number of colonies sampled at each locality

Thermal regime descriptors were compiled from weekly mean sea surface temperature data collected from IGOSS, Integrated Global Ocean Services System Products Bulletin (http://iridl.ldeo.columbia.edu/SOURCES/.IGOSS/) for quadrats of 1° longitude × 1° latitude and from 1982 to the year of sampling (2008–2009).

### DNA extraction

DNA extraction was performed using CTAB (cetyl trimethylammonium bromide)-based extraction method [37]. Briefly, coral tips were lysed 2 h in 600 μL CTAB buffer (2% CTAB, 0.2% β-mercaptoethanol, 1.4 M NaCI, 20 mm EDTA pH 8, I00 mm Tris-HCl pH 8, 100 μg/mL proteinase K) at 60 °C. An equal volume of chloroform:isoamyl-alcohol (24:1) was then added. After centrifugation for 10 min at 14,000*g*, the aqueous phase was transferred to a new tube and DNA was precipitated by adding 400 μL of ice-cold isopropanol and incubating 1 h at − 20 °C. After centrifugation for 15 min at 14,000*g*, the supernatant was discarded and the pellet washed with 70% ethanol. The pellet was air-dried for 5 min and resuspended in water.

### Host haplotype identification

The mitochondrial variable open reading frame (ORF) was amplified with FATP6.1 (5′-TTTGGGSATTCGTTTAGCAG-3′) and RORF (5′-SCCAATATGTTAAACASCATGTCA-3′) primers [38] and submitted to sanger sequencing (GenBank submission ID 2033564). Protein-coding sequences were analyzed using MEGA version 6 [39]. Sequence alignment was performed using MUSCLE. The best model (Kimura-2 parameters with uniform substitution rates) was selected for the lowest BIC (Bayesian Information Criterion). Maximum-likelihood tree was computed with the best model, and the robustness of the tree was tested with 1000 bootstrap replicates.

### 16S rRNA gene and internal transcribed spacer ITS2 metabarcoding

For all 94 individual samples, amplicon sequencing was performed for the two markers separately. For bacterial communities, 16S rRNA gene libraries were generated using the 341F (5′-CCTACGGGNGGCWGCAG-3′) and 805R (5′-GACTACHVGGGTATCTAATCC-3′) primers targeting the variable V3V4 loops [40]. For each sample, a first PCR amplification of 35 cycles was performed for the generation of amplicons (96 °C for 30 s, 55 °C for 30 s, 72 °C for 1 min) followed by 15 cycles with Illumina adaptors and sequencing index for thelibrary (95 °C for 15 s, 58 °C for 30s, 72 °C for 1 min).

*Symbiodinium* assemblages were analyzed using ITS2 (internal transcribed spacer of the ribosomal RNA gene) amplicon libraries with specific primers targeting a ~ 350 bp sequence: ITS2-F (5′-GTGAATTGCAGAACTCCGTG-3′) and ITS2-R (5′-CCTCCGCTTACTTATATGCTT-3′) [41, 42]. For each sample, a first PCR amplification of 35 cycles was performed for the generation of amplicons (96 °C for 30 s, 54 °C for 30 s, 72 °C for 1 min) followed by 15 cycles with Illumina adaptors and sequencing index for the library (95 °C for 15 s, 58 °C for 30 s, 72 °C for 1 min).

For both 16S rRNA gene and ITS2 markers, paired-end sequencing with 250 bp read length was performed on two different flow cells using the MiSeq system (Illumina) using the v2 chemistry according to the manufacturer’s protocol. Sequencing was performed at the McGill University in the Génome Québec Innovation Centre, Montréal, Canada. Raw sequence data are available in the SRA database (BioProject ID PRJNA393088, BioSample ID SUB2829485).

### Sequence analysis of metabarcoding datasets

The FROGS pipeline (Find Rapidly OTU with Galaxy Solution) implemented on a galaxy instance (http://sigenae-workbench.toulouse.inra.fr/galaxy/) was used for data processing [43]. In brief, paired reads were merged using FLASH [44]. After denoising and primer/adapters removal with cutadapt [45], de novo clustering was done using SWARM that uses a local clustering threshold [46], with aggregation distance *d* = 3 after denoising. Chimera were removed using VSEARCH [47]. We filtered the dataset for singletons and performed affiliation using Blast+ against the Silva database (release 128, September 2016) for 16S rRNA gene amplicons. For ITS2 metabarcoding, the *Symbiodinium* clade was assessed using blastn best hit against the nr/nt database of the NCBI [48]. To confirm blast identification, we performed phylogenetic analysis using MAFFT to produce sequence alignment and FastTree (GTR + CAT model) to compute tree with the approximately maximum-likelihood method. Finally, OTU tables were produced in a standard BIOM format for subsequent analyses.

### Statistical analyses

All statistical analyses were done using R version 3.3.1 ([49], http://www.R-project.org). We used the phyloseq R package for community composition analysis [50] to infer alpha diversity metrics at the OTU level, as well as beta diversity (between sample distance) from the OTU table. Community similarity was assessed by principal coordinate analysis (PCoA) using the Bray-Curtis dissimilarity index.

We performed non-parametric Kruskal-Wallis tests (since normality of residuals and homogeneity of variances were rejected (Shapiro and Bartlett test, respectively)) to compare alpha diversity metrics (Chao1 and Shannon). When Kruskal-Wallis tests were significant, we then computed pairwise comparisons between group levels (post hoc analyses) with Bonferroni corrections for multiple testing using Dunn tests.

Redundancy analysis [51] (hereafter named RDA) was used to investigate the variations of the different bacterial OTU/*Symbiodinium* clades under the constraint of the environmental variables. Bacterial OTUs and *Symbiodinium* clades were Hellinger-transformed before performing RDAs [52] on datasets of geography, temperature, and host genotypes (Additional file 1: Table S1). Then significant variables (i.e., variables that significantly explained changes in the distribution of OTUs/clades) were identified using a forward-selection procedure (999 permutations), implemented in the R “vegan” package and in the “rda” and “ordiR2step” functions. For all analyses, the threshold significance level was set at 0.05.

## Results

### Sampling sites display diverse and contrasted thermal regimes

**Fig. 1:**
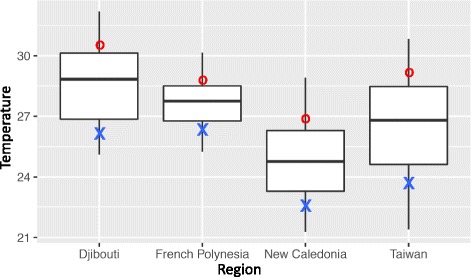
Temperature distribution for the four sampled regions. The boxplots represent the lower and upper quartiles, the black lines represent the mean temperatures, and the whisker ends represent the minimal and maximal temperatures for each region. Blue crosses indicate the yearly mean temperatures of the three colder months, and red dots indicate the yearly mean temperatures of the three warmer months

To have a precise view of the thermal regimes in the different sampling sites, we extracted several descriptors over a long-term continuous monitoring (weekly) of mean sea surface temperature (SST) data. We computed the annual mean temperature and the minimum and maximum temperatures, as well as mean temperatures, for the three warmer or colder months over these time-series records and annual temperature variance (Table 2, Fig. 1). We had very contrasted thermal regimes between the sampled regions with high and low annual variations in Taiwan and French Polynesia, respectively. The minimal temperatures were lower in New Caledonia and Taiwan compared to Djibouti and French Polynesia. The maximum temperatures were less contrasted with a more continuous increase from New Caledonia to French Polynesia, then Taiwan and Djibouti.

**Table 2:** Thermal regime descriptors of the four sampled regions calculated from weekly SST data (from 1982 to the year of sampling, 2008 or 2009, according to regions)

	Djibouti	French Polynesia	New Caledonia	Taiwan
Mean temperature (°C)	28.60	27.67	24.77	26.56
Max temperature (°C)	32.20	29.95	28.78	30.83
Min temperature (°C)	25.11	25.25	21.28	21.40
Mean of the warmer months (°C)	30.46	28.75	26.82	29.14
Mean of the colder months (°C)	26.32	26.51	22.81	23.88
Variance (°C)	3.17	1.05	3.04	4.56

### *Pocillopora damicornis sensu lato* morphotypes corresponded to two different clades

Analysis of mitochondrial ORF for all samples revealed two clades corresponding to *Pocillopora* types 3c, 3e, 3g, 3h, and 7a (clade 2) for all samples from Djibouti and *Pocillopora* type 5 (clade 1) for samples from New Caledonia, Taiwan, and French Polynesia [33] (Additional file 2: Figure S1 and Additional file 1: Table S1). This is consistent with the known geographical distribution of *Pocillopora* lineages with a wide distribution of clade 1, i.e., types 4 and 5, namely *Pocillopora damicornis* and *Pocillopora acuta*, respectively, throughout the Pacific Ocean, and the presence of types 3 and 7 (*P. damicornis sensu lato* morphotype) in the Western Indian Ocean [32, 34].

### *Symbiodinium* assemblages revealed high specificity as well as high background diversity

The relative proportions of *Symbiodinium* types in each coral sample were analyzed using high-throughput sequencing method. ITS2 amplicon sequencing with MiSeq yielded a total of 4,076,779 informative clusters. After clustering and filtering for OTUs containing less than 100 sequence tags, 53 clusters of ITS2 were obtained, corresponding to 3,990,373 sequences (Additional file 3: Table S2). Taxonomic affiliation was performed using blastn in comparison to NT followed by phylogenetic analysis. Maximum-likelihood trees confirmed the presence of clades A1, C1, C3, C15, D1, and G with largely unresolved polytomies in the clade C (Additional file 4: Figure S2), such as observed previously [53]. Nevertheless, the topology is congruent with previous works on the molecular taxonomy of *Symbiodinium* [54].

**Fig. 2:**
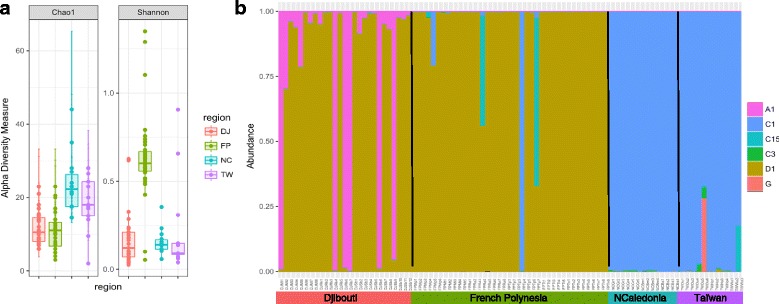
*Symbiodinium* diversity: **a** Alpha diversity (Chao1 and Shannon) index comparison between samples from the four regions based on ITS2. *DJ* Djibouti, *FP* French Polynesia, *NC* New Caledonia, *TW* Taiwan. **b** Proportion of *Symbiodinium* sub-clades per sample

**Fig. 3:**
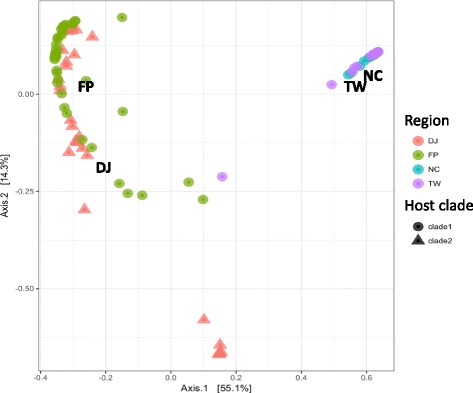
Principal coordinate analysis of Bray-Curtis dissimilarities (ITS2) between all pairs of samples (colored by region of origin and shaped by *Pocillopora* host haplotype). *DJ* Djibouti, *FP* French Polynesia, *NC* New Caledonia, *TW* Taiwan. These axes represent the two synthetic variables explaining the most variation of the dataset

OTU richness assessed by Chao1 index was significantly different between sites (Kruskal-Wallis test, *χ*^2^ = 36.2, df = 3, *p* < 0.0001) and particularly slightly higher in New Caledonia and Taiwan (see Additional file 5: Table S3). Moreover, Shannon diversity index was significantly different between sites (Kruskal-Wallis test, *χ*^2^ = 58.9, df = 3, *p* < 0.0001) and particularly higher for French Polynesia compared to the others regions (Fig. 2a) (see Additional file 5: Table S3). Only one *Symbiodinium* clade was highly dominant per sample (accounting for more than 95% of sequences) (Fig. 2b), suggesting high specificity for each association. In New Caledonia and Taiwan, *Symbiodinium* clade C1 was almost exclusively present in all samples. Clade D1 was dominant in French Polynesia samples (except for two samples), whereas a more diverse pattern is observed in Djibouti with a majority of clade D1 as well as a substantial proportion of clade A1, either as the main *Symbiodinium* clade or in association with D1. Accordingly, principal coordinate analysis based on Bray-Curtis dissimilarity index followed this partitioning with two main groups (French Polynesia and Djibouti vs. Taiwan and New Caledonia) separated along the first axis (which explained 56.1% of the total variation) (Fig. 3). Samples from Djibouti forming a separate group along the second axis are hosting *Symbiodinium* clade A1. Samples from different host clades were overlapping on both axes.

### Bacterial microbiota was highly diverse with a small number of core taxa

**Fig. 4:**
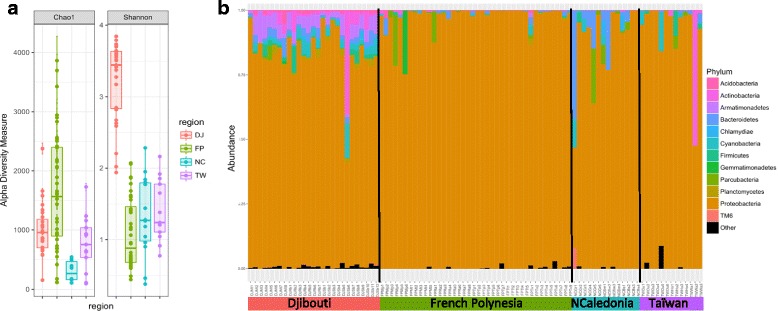
Bacterial diversity: **a** Alpha diversity (Chao1 and Shannon) index comparison between samples from the four regions based on 16S rRNA gene. *DJ* Djibouti, *FP* French Polynesia, *NC* New Caledonia, *TW* Taiwan. **b** Phylum-level community composition per sample

The bacterial microbiota of each coral sample was analyzed using 16S rRNA gene (V3V4) amplicon sequencing with MiSeq. It yielded a total of 8,198,530 informative clusters. After singleton filtering, we obtained 33,649 OTUs (representing a total of 7,108,067 sequences) that could be annotated using the Silva 16S database. After filtering for chloroplast and non-bacterial sequences, we obtained 31,076 OTUs (Additional file 5: Table S3) representing 6,569,797 sequences. Notably, the 100 most abundant OTUs represented more than 90% of the whole sequences. OTU richness assessed by Chao1 index was the highest in French Polynesia and followed by a slightly higher level in Djibouti compared to samples of New Caledonia and Taiwan (Fig. 4a, Additional file 6: Table S4). Moreover, Shannon diversity index was significantly higher for Djibouti compared to the others regions. These results indicated a high number of rare OTUs in French Polynesia and higher evenness in Djibouti.

Phylum-level assignment of bacterial OTUs indicated the dominance of Proteobacteria in all samples (Fig. 4b). Within this phylum, the majority of sequences were affiliated to the genus *Endozoicomonas* (family Hahellaceae) representing 66.9% of the overall sequences (Additional file 7: Table S5). Three other genera, *Arcobacter*, *Acinetobacter*, and *Sphingomonas* were present at appreciable relative proportions (6.6, 1.9, and 1%, respectively, of total phyla tags). *Arcobacter* (Campylobacteraceae) was particularly abundant in samples from Djibouti (25% of sequence tags), whereas *Acinetobacter* (Moraxellaceae) represented 9.4% of the sequence tags in New Caledonia.

Among 227 families and 513 genera in the whole dataset, only the genus *Endozoicomonas* was common to all samples. We also considered as core phylotypes (at the family and genus level) the taxa shared by 50% of the individuals within each region (Table 3). Two additional genera, *Arcobacter* and *Acinetobacter*, fall into this category in addition to *Endozoicomonas*. At the family level, four supplementary families, namely Comamonadaceae, Rhodobacteraceae, Caulobacteraceae, and Sphingomonadaceae, were shared by at least 50% of samples. In all cases, core taxa were among the 10 most abundant in the whole dataset (14 families and 6 genera were shared by at least 50% of the overall samples) (Additional file 7: Table S5).

**Table 3:** Number of samples for each region for core taxa (shared by at least 50% of samples within each region and 50% of the overall samples) at the family and genus levels

Rank	Taxa	DJ (27)	FP (40)	NC (14)	TW (13)	Total (94)
Family	**Hahellaceae**	27	40	14	13	94
	**Moraxellaceae**	25	37	13	12	87
	Comamonadaceae	26	36	13	10	85
	**Campylobacteraceae**	25	34	13	12	84
	Rhodobacteraceae	25	36	11	9	81
	Caulobacteraceae	24	30	9	7	70
	Sphingomonadaceae	23	26	9	8	66
Genus	*Endozoicomonas*	27	40	14	13	94
	*Acinetobacter*	26	35	13	12	86
	*Arcobacter*	27	33	12	12	84

The total number of samples for each region is indicated into brackets. The family ranks also correspond to the core genus are indicated in bold

Considering each region independently, strikingly much more taxa were shared between at least 50% of samples from Djibouti (27 families and 46 genera) (Additional file 7: Table S5), which could be linked to higher equitability. Twelve families and 5 genera were core to French Polynesia samples, 12 families and 4 genera for New Caledonia, whereas only 7 families and 3 genera were shared by at least 50% of samples from Taiwan (Additional file 7: Table S5).

**Fig. 5:**
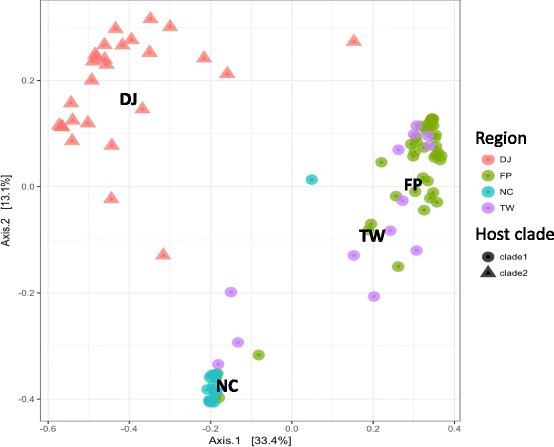
Principal coordinate analysis of Bray-Curtis dissimilarity index (16S rRNA gene) between all pairs of samples (colors and shapes indicate region of origin and *Pocillopora* host haplotype, respectively). Axes represent the two synthetic variables explaining most variation in the dataset (about 46%)

Lastly, principal coordinate analysis based on Bray-Curtis dissimilarity index showed a partitioning of bacterial communities by both host haplotype and region (Fig. 5). Samples from Djibouti (*Pocillopora* clade 2) and New Caledonia (*Pocillopora* clade 1) formed separate groups, whereas Taiwan and French Polynesia samples (clade 1) overlapped on the two principal axes.

### *Symbiodinium* communities were mostly influenced by minimal temperatures, whereas bacterial community structure was associated to host haplotype and mean temperatures

**Fig. 6:**
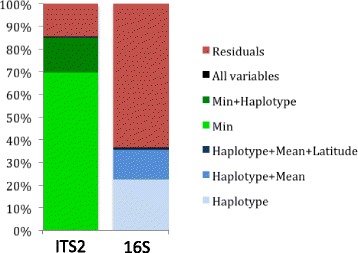
Cumulative variations explained by the addition of significant variables for the reduced models identified using RDA and forward selection procedures. *Min* minimal temperature, *Mean* mean temperature, haplotype: coral haplotype

To disentangle the influence of biogeography, temperature, and host haplotype (Additional file 1: Table S1) on coral communities of both *Symbiodinium* and bacteria, we computed redundancy analysis (RDA) and forward selection procedures. First, we performed RDAs on the whole dataset (geography, thermal regime, and host haplotype). A very high proportion (85%) of *Symbiodinium* clade distribution was explained by the variables used in this study; this proportion was lower although important (36%) for the bacterial OTU distributions (Fig. 6, Additional file 8: Table S6). Forward selection procedures were carried-out on the whole dataset to identify the significant variables (Additional file 1: Table S1) constraining the distribution of both communities. This study highlighted (i) that minimal temperature followed by host haplotype mostly constrained *Symbiodinium* distribution in the 94 samples (*p* = 0.001) and (ii) that in contrast, host haplotype followed by mean temperature then latitude mostly constrained bacteria distribution (*p* = 0.001).

## Discussion

### Diversity of *Pocillopora damicornis sensu lato* samples

In this study, we sampled *Pocillopora damicornis sensu lato* colonies in four regions and phylogenetic analyses revealed a complex pattern. Samples from French Polynesia, New Caledonia, and Taiwan corresponded to *Pocillopora* clade 1 (more precisely type 5, newly assigned to *P. acuta* sensu) [33, 34]. Samples from Djibouti corresponded to *Pocillopora* clade 2 (types 3 and 7) later assigned to SSH12 and SSH13 and all consistent with *P. damicornis* morphotype [32]. Morphological characters have been shown to be insufficient to discriminate species in the *P. damicornis* complex, and genetic markers are necessary to discriminate the species [33]. This species complex displays in addition high phenotypic plasticity and cryptic lineages [32]. Nevertheless, as we used a single mitochondrial marker for *Pocillopora* clade identification, we cannot completely rule out the possibility of mitochondrial introgression or hybridization that seem to be common within this genus [55, 56]. Finally, although it was proposed that *Symbiodinium* assemblages may be useful for integrative taxonomy of *Pocillopora* species [57], we found in this study that *Symbiodinium* communities did not discriminate *Pocillopora* haplotypes.

### Diversity of coral microbiota

The core microbiome can be broadly defined as the stable and consistent taxonomic groups associated to a particular habitat [58]. These commonly associated microbial communities are likely of ecological and functional importance in the holobiont fitness. The definition of the core microbiome in corals is variable among authors, ranging from 30 to 100% of shared bacterial phylotypes at all different taxonomic levels from kingdom to strain (reviewed in [3]). These studies already highlighted the high diversity and variability of microbial communities in corals. We defined here the core microbiome as taxa (OTUs/genera/families) present in at least 50% of samples within each population and thus reflecting stable associations with *P. damicornis sensu lato*.

All core taxa of bacteria were among the most abundant in the dataset, which is in contradiction with previous study identifying rare taxa as ubiquitous endosymbionts [59]. Bacteria of the *Endozoicomonas* genus were present in all samples and were the most abundant in the whole dataset, making up from 33.3% of the total number of sequence tags in Djibouti (consistent with a higher evenness) to 87.4% in French Polynesia where rare OTUs were the most diverse. This group has been described for the first time only recently in marine slugs [60]. So far, they have been identified in numerous coral species and are now considered as ubiquitous endosymbionts of many marine host species (see [61] for a review). Notably, comparative genomic analysis of different strains of *Endozoicomonas* from different hosts suggests a common role in carbohydrate cycling with potential specificities in amino-acid synthesis [62]. In addition, *Acinetobacter* and *Arcobacter* were respectively present in 86 and 84 samples over 94 and were the third and second most abundant genus after *Endozoicomonas*. Although considered as a terrestrial bacteria, *Acinetobacter* (gamma-proteobacteria) can be dominant in bleached corals [63]. It has also been identified in healthy corals where it can be abundant [64–66], but its function remains elusive. Bacteria of the genus *Arcobacter* (epsilon-proteobacteria) are associated to a wide range of habitats, as free-living or pathogenic, and are especially abundant in marine environments [67] and have been identified specifically in diseased corals in some studies [68], sometimes also in healthy samples [69], and has been show to increase during pathogenesis [70, 71]. *Arcobacter* has also been found in high abundances in necrotic sponges [72] as well as moribund oysters [73] where it is associated with decreased bacterial diversity and may act as an opportunistic pathogen. Although detectable at background abundances in the majority of samples, *Arcobacter* was highly abundant in all samples from Djibouti (25% of sequence tags vs. less than 1% in the other regions), indicating that this genus can also be abundant in healthy hosts and moreover associated with high bacterial diversity.

Metabarcoding targeting ITS2 provides highly sensitive measurements of *Symbiodinium* diversity and relative proportions [42]. It was used with 454-pyrosequencing [74, 75] and more recently with Illumina MiSeq sequencing [76]. Although we did not find ubiquitous clades for the whole samples, we showed using MiSeq sequencing striking differences in terms of *Symbiodinium* associations between populations. Clade D1 was dominant in French Polynesia. This is consistent with a recent study in Moorea that showed association with *Symbiodinium* clade D and few clade C [77]

Colonies from Djibouti were also mainly associated with clade D1, while clade C1 was dominant in Taiwan and New Caledonia. To our knowledge, this is the first study of symbiotic communities associated with *Pocillopora* in Djibouti, New Caledonia, and Taiwan. A comparative study of *Symbiodinium* clades associated with scleractinian corals from the Persian Gulf with annual temperatures from 16 up to 36 °C revealed the dominance of clade C3 associated with a lower proportion of A1 and minor quantities of C15, whereas clade D was not detected [78].

Even if one single clade was dominant in almost all samples, we detected background abundances of the other clades in all regions constituting the so called “*Symbiodinium* rare biosphere” [74]. The cryptic diversity that we reported here is actually very high (H′ Shannon index up to 0.6 for French Polynesia samples, Fig. 2a) and falls into a similar range such as that reported in the latter work for three Pocilloporid species [79]. Such high diversity can potentially confer higher adaptive potential to their hosts through redundant or complementary symbiotic functions and/or potential for symbiont switching or shuffling [80]. High-throughput methods (ITS metabarcoding using 454) applied to the generalist and environmentally sensitive *Acropora* species also revealed the same pattern across Western Australia with clade C being dominant in all samples with background abundances of other clades [75].

Finally, bacterial communities in corals may also be shaped by the presence and type of photosymbionts [81] through the use of DMSP released by the algae [82], but at a finer scale, we could not find any relationship for the same host species between *Symbiodinium* type and bacterial communities as was also the case in *Seriatopora hystrix* [28]. In addition, we observed contrasting patterns in terms of alpha diversity between the *Symbiodinium* and bacteria communities, with notably an opposite pattern on the Shannon index for samples from Djibouti (highest Shannon index for 16S, Fig. 2a) and French Polynesia (highest Shannon index for ITS2, Fig. 4a). Previous work in *Isopora palifera* at the seasonal scale also revealed important variations in Shannon diversity that are uncoupled from changes in *Symbiodinium* community composition [64].

### Influence of geography on coral microbiota is very low

Because we found different microbiota composition between samples, we used redundancy analyses in order to identify variables that significantly constrained their composition. We first found that the influence of geography was low, since longitude had no significant effect and the effect of latitude in our dataset was null for *Symbiodinium* assemblages and was marginal on bacterial communities (less than 1% when added to the variance explained by host haplotype and mean temperature). In contradiction with this result, geographic clustering of symbionts is expected to be high in brooding species with vertical transmission, compared to those with horizontal transmission that would in contrary favor propagation of locally adapted symbionts. *Pocillopora damicornis* is a hermaphrodite brooder [83, 84] thus transmitting its symbionts vertically [85] and is able to release sexual as well as parthenogenetic larvae [86, 87]. However, these results are similar to others studies showing that bacterial communities associated with different coral species are stable through space and time [2]. Thus, we hypothesize that low parental effect on symbiont communities may be explained by the relatively high connectivity and long-range dispersal for this species compared to other brooders [35, 88], which would mitigate the effect of geography and allow higher symbiont mixing with the local microbial environment.

### Influence of host haplotype is higher for bacterial communities than for *Symbiodinium*

Although we did not find a significant link between microbiota and geography, we highlighted links with host haplotypes, particularly for bacterial microbiota. As the two host clades sampled in this study were from potentially different microbial environments, we cannot rule out a confounding effect between host haplotype and environmental communities present in the seawater at the time of larval settlement and bacterial microbiota acquisition. Nevertheless, others studies observed that host genotypes might influence bacterial microbiota, since (i) different coral species harbored different microbiota composition [2] and (ii) differential specificity between closely related corals and abundant *Endozoicomonas* endosymbionts were found across global scales [89].

In contrast, we found a significant but less important link between *Symbiodinium* and host haplotype. This observation might also be linked to the adaptive bleaching hypothesis, which proposes that coral bleaching is an adaptive mechanism through the modification of *Symbiodinium* composition, with acquisition of clades with higher thermal tolerances [90]. For example, endosymbiotic flexibility (the ability of a coral species to associate with multiple *Symbiodinium* clades) was observed to be linked with resistance [15]. In addition, *Pocillopora* is a generalist host, displaying a high intraspecific and interspecific flexibility in terms of *Symbiodinium* assemblages (mainly among clades A, C, and D) [91].

### Influence of temperature is higher for *Symbiodinium* than for bacterial microbiota

Although host genotypes mostly constrained bacterial assemblages, we also found a significant link with mean temperatures. Interestingly, reciprocal transplants of *Acropora* corals at a reduced geographic scale revealed as well that their microbiome were different across thermally variable habitats and changed over time after transplantation [76]. As a consequence, bacterial communities might be linked to heat tolerance of their coral hosts, but further studies of their dynamics during a thermal stress are needed to understand their contribution to the holobiont response.

Strikingly, redundancy analysis revealed that most variation in *Symbiodinium* communities was actually explained by annual minimal temperatures (70%), whereas we observed no significant correlation with mean, maximal, or annual fluctuations of temperatures. In *Acropora*, it has been shown experimentally that *Symbiodinium* type alters larvae settlement in a temperature-dependent fashion [92]. In the latter work, lower temperatures and not higher temperatures adversely affected recruitment by reducing larval survival and settlement. Accordingly, we propose that host/*Symbiodinium* associations are stable in space at a regional scale and are actually more sensitive to a minimal, threshold temperature, than to annual variations or to maximal temperatures.

In particular, clade D is common in all areas colonized by scleractinian corals [93], and many studies have shown that it confers higher thermotolerance to its host through photoprotection [94, 95] and that it increases in proportion after bleaching events [20, 95, 96]. Clade D is thus commonly considered as stress resistant and potentially opportunistic for compromised corals facing stressful conditions [97]. Nevertheless and in accordance to the link we observed with minimal temperatures, some studies have revealed that cold stress may be detrimental for coral harboring this symbiont [98, 99]. More recently, it has been shown experimentally that clade D1a suffered more photodamages at low temperatures than clade C3 [100]. Thus, it seems that clade D may not display higher plasticity in terms of thermal range, but rather that it is only adapted to hot thermal environments. Because climate changes will not only be associated with global increase of sea surface temperatures, but also to extreme thermal events including cold episodes [101], further studies are required to understand the potential response of coral reef in the future, particularly for regions with highly fluctuating thermal regimes and low temperature thresholds.

## Conclusions

The hologenome theory of evolution reboots elements of the Lamarckian evolution and has been thus a matter of much debate (reviewed in [7, 102]). Although the host genome follows a Mendelian framework, potential changes in microbial assemblages may be inherited to the next generation. Conversely, for the selection to operate on holobiont phenotypes at evolutionary scales, specificity of the interaction through coevolution between partners would be expected.

Using combined high-throughput barcoding approaches for both bacterial and *Symbiodinium* communities, we showed that variation in *Symbiodinium* composition is mostly explained by thermal regime, especially minimal temperatures, whereas bacterial communities are much less related to temperature modifications. In this context, we propose that *Symbiodinium* types might confer more enhanced adaptive capacities to temperature modifications than the bacterial microbiota. However, *Symbiodinium* clade D was known to be adapted to high temperature, and we found a negative relationship with low temperatures, suggesting low plasticity for this clade. Such low plasticity might limit the adaptive capabilities of a coral associated to clade D and living in highly variable thermal regime. However, a high background diversity of *Symbiodinium* was also observed, providing the potential for coral colonies to adapt or acclimatize to future environmental changes via symbiont shuffling (i.e., changes in the relative proportion of *Symbiodinium* types constituting the within host community).

Our study may thus contribute to new insights into the importance of microbial (i.e., bacterial and *Symbiodinium*) communities for holobiont functioning as well as the relative importance of host and environmental factors in shaping the interaction.

## Additional files

Additional file 1: **Table S1.** Sample metadata including geographic and abiotic variables (temperature descriptors) as well as *Pocillopora* haplotype identification. (XLSX 55 kb)

Additional file 2 **Figure S1.** Maximum-likelihood tree of the mitochondrial ORF-defining *Pocillopora* types. Numbers are bootstraps (%) reflecting clade support. (PPTX 307 kb)

Additional file 3: **Table S2.** *Symbiodinium* OTU table with sequence tag counts per sample and taxonomic affiliation (XLSX 62 kb)

Additional file 4: **Figure S2.** Maximum-likelihood tree of the 53 *Symbiodinium* OTUs based on ITS2, together with GenBank representatives of each identified clade. Numbers are bootstraps (%) reflecting clade support. (PPTX 438 kb)

Additional file 5: **Table S3.** Bacterial OTU table with sequence tag counts per sample and taxonomic affiliation. (XLSX 49 kb)

Additional file 6: **Table S4.** Diversity indices calculated on bacterial diversity for each sample and statistical analyses of differences between regions. (XLSX 136 kb)

Additional file 7: **Table S5.** Number of bacterial sequences and positive samples for each region at the family and genus levels. Taxa shared by at least 50% of samples in one population are colored in light gray, whereas taxa shared by at least 50% of the overall samples are colored in dark gray. We considered as core microbiota the taxa shared by 50% of samples for each of the four populations (regions) studied. (XLSX 128 kb)

Additional file 8: **Table S6.** Variations in bacterial (16S rRNA gene) and *Symbiodinium* (ITS2) communities explained by the addition of significant variables for the reduced models identified using RDA and forwardselection procedures. (XLSX 38 kb)
