# Supplementary figures and images for: Thermal regime and host clade, rather than geography, drive Symbiodinium and bacterial assemblages in the scleractinian coral Pocillopora damicornis sensu lato
Source: Microbiome. 2018 Feb 20;6:39. doi: 10.1186/s40168-018-0423-6 (PMC5819220; doi:10.1186/s40168-018-0423-6)

## Slide 1
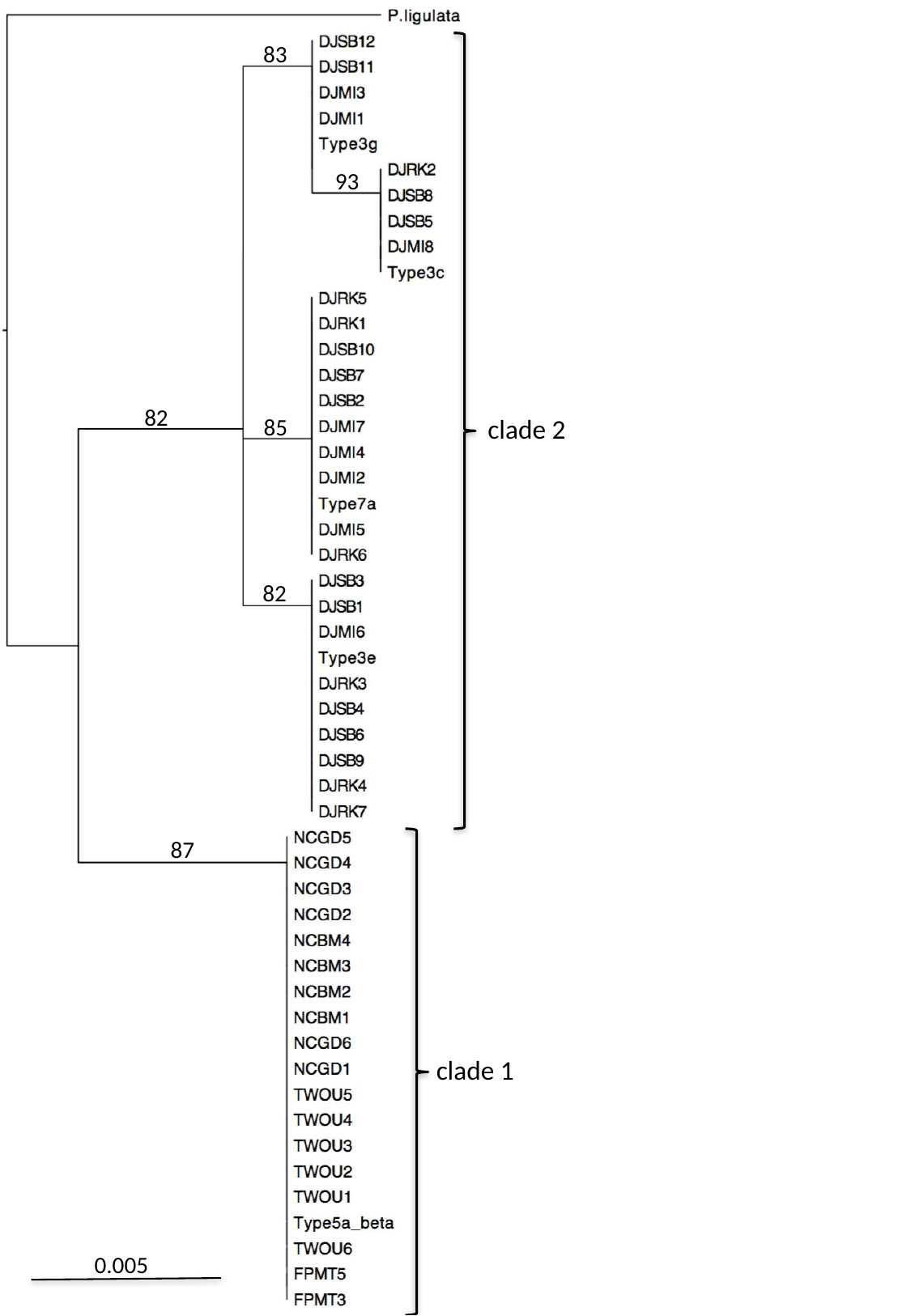

83
93
82
85
82
87
clade 2
clade 1
0.005

Supplement: Supplementary file 2 — Figure S1. Maximum-likelihood tree of the mitochondrial ORF-defining Pocillopora types. Numbers are bootstraps (%) reflecting clade support. (PPTX 307 kb) [file 40168_2018_423_MOESM2_ESM.pptx]

## Slide 1
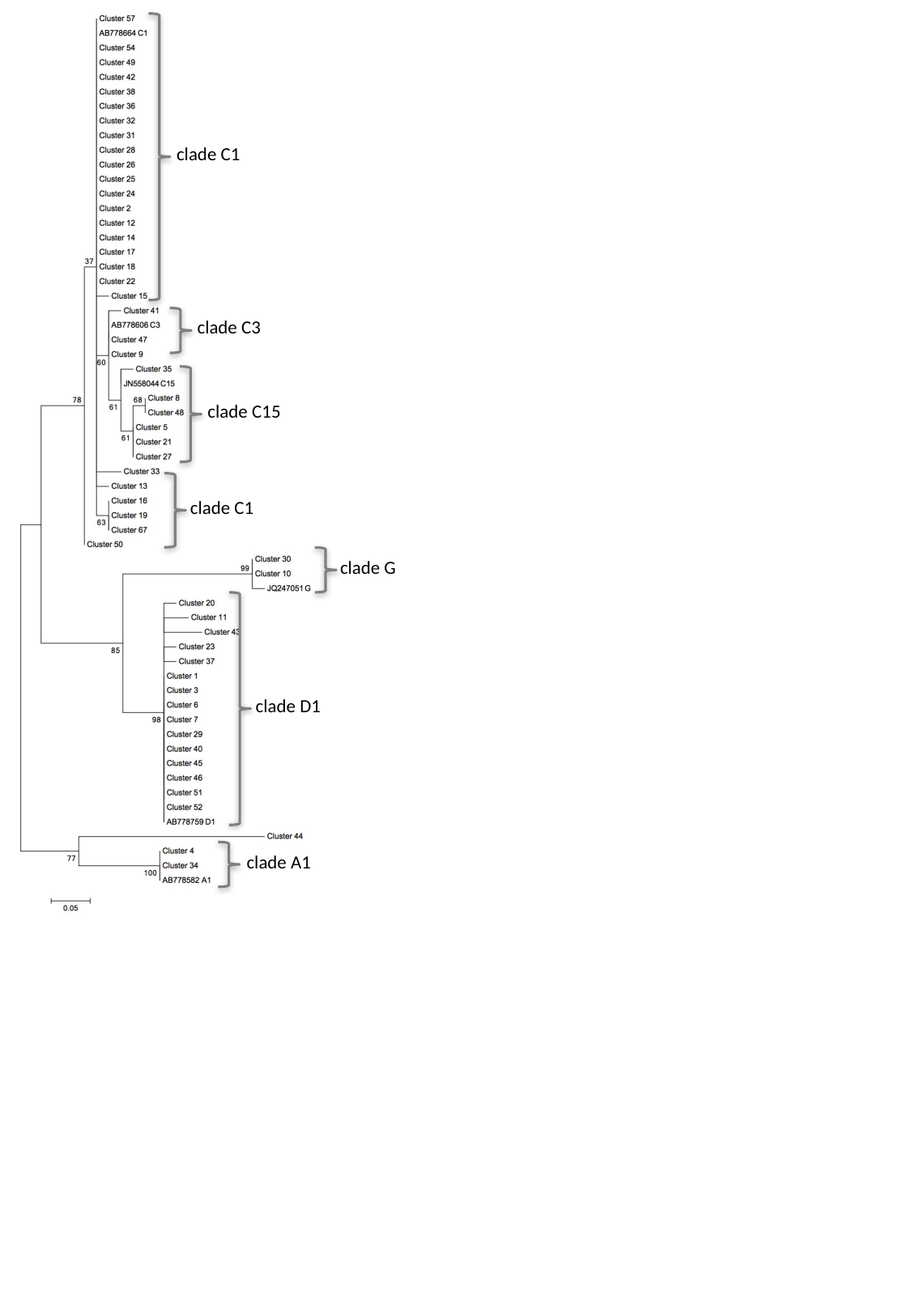

clade C1
clade C3
clade C15
clade C1
clade G
clade D1
clade A1

Supplement: Supplementary file 4 — Figure S2. Maximum-likelihood tree of the 53 Symbiodinium OTUs based on ITS2, together with GenBank representatives of each identified clade. Numbers are bootstraps (%) reflecting clade support. (PPTX 438 kb) [file 40168_2018_423_MOESM4_ESM.pptx]
